# Degenerated huge retroperitoneal leiomyoma presenting with sonographic features mimicking a large uterine leiomyoma in an infertile woman with a history of myomectomy: a case report

**DOI:** 10.1186/1752-1947-5-578

**Published:** 2011-12-16

**Authors:** Amr A Soliman, Bassma ElSabaa, Noha Hassan, Hassan Sallam, Tarek Ezzat

**Affiliations:** 1 Department of Obstetrics and Gynaecology, University of Alexandria, Egypt; 2 Department of Pathology, University of Alexandria, Egypt; 3 Department of Surgery, University of Alexandria, Egypt; 4 Division of Surgery and Interventional Science, University College London, London, UK

## Abstract

**Introduction:**

Retroperitoneal leiomyomata are rare. They are either mistaken preoperatively for malignant retroperitoneal tumors or dealt with as cases of subserous leiomyomata that turn out intraoperatively to be huge retroperitoneal masses of unknown nature.

**Case presentation:**

We report the case of a 46-year-old nulligravid female patient of Middle Eastern ethnicity who presented to our university hospital with lower abdominal as well as pelvic pain along with a bloated sensation. She also reported noticing an unusual increase in her abdominal girth. These symptoms developed over the previous two months. Preoperative investigation by means of an ultrasound suggested a degenerated subserous huge uterine leiomyoma. An abdominal hysterectomy was planned. Intraoperatively, a normal sized uterus was found, the surface of which was studded with multiple variable sized pedunculated subserous leiomyomata. Another huge retroperitoneal soft to firm mass was found extending from her left pelvic wall to the level of her spleen, with no connections to her uterus. The mass was excised and a histopathological examination revealed a degenerated leiomyoma.

**Conclusion:**

Some unusually located extra-uterine leiomyomata have been reported; retroperitoneal leiomyoma being among them. The origin of such tumors is still obscure; a parasitic origin as well as Müllerian cell rests or smooth muscle cells in the retroperitoneal vessels wall have been suggested. An 'iatrogenic' origin for such growths is also a possible theory. The origin of uncommonly located leiomyomata is an unexplored issue that merits more investigation.

## Introduction

Retroperitoneal leiomyomata are a rare occurrence. Kang *et al. *found only 46 reported cases during their PubMed search up to October 2008 [1]. We updated this search using the keywords 'extra-uterine', 'leiomyoma' and 'retroperitoneal leiomyoma' up to September 2011 to find only four more reported cases in that time period [2-5]. This rarity makes it an unexpected incident that is either mistaken preoperatively for a retroperitoneal mass which could be malignant or, as in our case, dealt with as a huge leiomyoma that presents intraoperatively as a huge retroperitoneal mass of unknown nature.

## Case presentation

**Figure 1:**
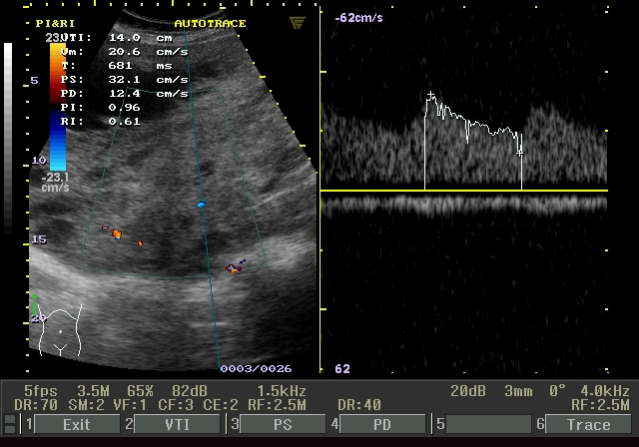
**US and Doppler examination of the leiomyoma**. US examination showing a heterogeneous echotexture of the mass and presence of multiple echo free areas, Doppler blood flow indices in and around the huge leiomyoma recorded a resistance index of 0.61 and pulsatility index of 0.96

The currently reported case is of a 46-year-old nulligravid female patient, of Middle Eastern ethnicity, who presented to the outpatient Gynaecology Clinic of our hospital one year ago. Her complaints included mild pelvic pain requiring the intake of non-steroidal anti-inflammatory drugs once a day two days a week at most. She also complained of pelvic heaviness combined with a gastrointestinal bloating sensation that required the daily intake of antiflatulent medication, which was not helpful in soothing the complaint. She had a past medical history of primary infertility for 20 years. She also had an abdominal myomectomy through a low-transverse abdominal incision 17 years earlier. A clinical examination revealed a huge pelvi-abdominal mass extending up to the level of her xiphisternum. A combined abdominal and vaginal ultrasound (US) revealed the presence of multiple leiomyomata with a huge subserous leiomyoma showing evidence of degeneration and extending to the level of the left lobe of her liver (Figure 1). There was no evidence of ureteric obstruction or renal pelvic ectasia as shown by a renal US. For an abdominal US we used a curvilinear probe with a frequency of 5 MHz, while for the transvaginal US a 7.5 MHz probe was used. The long history of the mass and its slow growth rate constituted a low index of suspicion of a uterine sarcoma.

**Figure 2:**
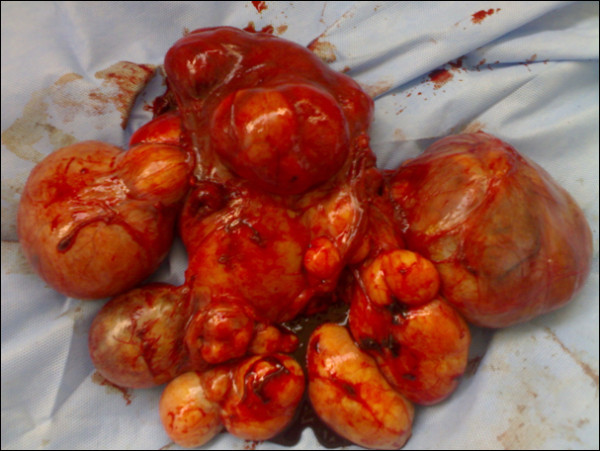
**Uterine fibroids**. Normal sized uterus with multiple subserous fibroids.

No further imaging investigations were requested due to the highly suggestive characteristics of the mass on US; accordingly the clinical diagnosis of a huge subserous uterine leiomyoma was made. An abdominal hysterectomy via a lower midline incision with left periumbilical extension was performed. Intraoperatively, the uterus was found to be of normal size with numerous variable sized pedunculated subserous leiomyomata diffusely attached to its surface (Figure 2).

**Figure 3:**
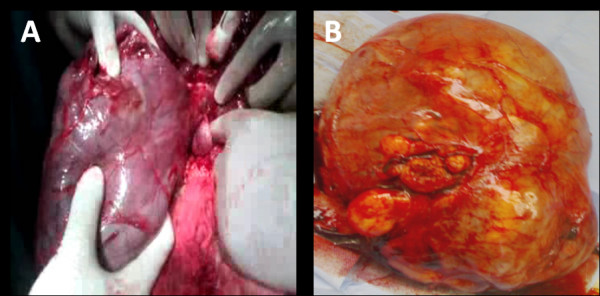
**Retroperitoneal leiomyoma**. Large 18 cm × 23 cm retroperitoneal fibroid completely separable from the uterine fibroids. **(A) **Intraoperatively and **(B) **after resection.

The huge degenerated leiomyoma previously delineated on sonography (Figure 3) turned out to be a retroperitoneal mass extending from the left side of her pelvis through the infundibulopelvic ligament upwards to the lower border of her spleen, with no connections with the leiomyomata-studded uterus. The mass displaced her mesosigmoid and her descending colon medially and even the root of the mesentry was displaced towards the midline. General surgeons were involved; they dissected the mass from its retroperitoneal vascular connections. The mass was in close proximity to her descending colon which was reflected medially in order to gain better access to the mass. The mass was crossing her left kidney anteriorly to the level of her spleen. Dissection in this area was very meticulous due to the proximity to the tail of her pancreas. The hysterectomy specimen and the huge retroperitoneal mass were sent for pathological examination. The postoperative course of our patient was very smooth and she was safely discharged on the fourth postoperative day. She returned ten days later for wound care which showed very good healing.

**Figure 4:**
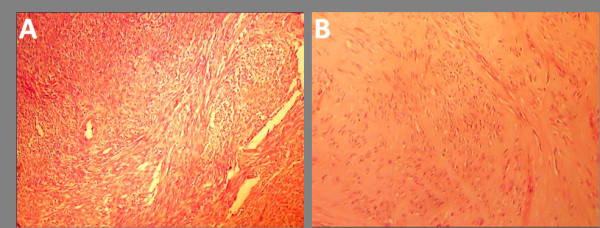
**Histological examination of the leiomyoma**. The retroperitoneal mass showed a whorled (fascicular) pattern of smooth muscle bundles separated by well vascularized connective tissue; smooth muscle cells were elongated with eosinophilic or occasional fibrillar cytoplasm and distinct cell membranes. **(A) **The growth was not infiltrative and contained thick walled arteries throughout and cleft-like spaces. **(B) **Areas of hyaline degeneration were also seen.

The histopathologic examination revealed a non infiltrative growth with scant mitotic activity (one mitotic figure per 10 high power field) with no atypia, thus confirming the benign leiomyomatous nature of this huge retroperitoneal growth, with evidence of hyaline degeneration (Figure 4)

## Discussion

Uterine leiomyoma is the most common benign gynecological tumor affecting as many as 25% of women in the reproductive age group [6], and is present in about 80% of all hysterectomy specimens [7]. In addition to the traditional patterns of leiomyomatous growth in the uterus, some unusual extra-uterine growth presentations are mentioned in the literature; benign metastasizing leiomyoma, disseminated peritoneal leiomyomatosis, intravenous leiomyomatosis, parasitic leiomyomata and retroperitoneal growth [8]. The incidence of retroperitoneal leiomyomata is quite low, and it is even lower for those extending to or originating in the abdomen. Of the reported retroperitoneal leiomyomata, 73% are located in the pelvis [9]. Most of the published case reports diagnosed the cases clinically as retroperitoneal growths with high suspicion of malignancy without suspecting their leiomyomatous nature [10-15]. The origin of such tumors is a puzzling issue with much scientific debate. Poliquin and coworkers observed a 40% association of retroperitoneal leiomyomas with uterine counterparts or a history of hysterectomy due to uterine leiomyomata [9]. Zaitoon suggested the parasitic theory for such tumor growth [10] while Stutterecker *et al. *claimed that Müllerian cell rests or smooth muscle cells in the retroperitoneal vessels wall are the putative origin [12]. Kho and Nezhat proposed an 'iatrogenic' origin for such growths while analyzing a case series of extra-uterine leiomyomata, mostly of retroperitoneal or intraperitoneal location with no visible connection to the uterus. They found out that 83% of their case series had previous abdominal operations, and 67% had myomectomies, most of them via laparoscopy with morcellation [16]. In our reported case, although the iatrogenic theory for such leiomyomata could explain the growth of the numerous pedunculated subserosal leiomyomata scattered on the uterine surface (Figure 2), it cannot explain the growth of the retroperitoneal mass which was completely separable from the uterus and seated deep in the posterior abdominal wall peritoneum. The concept suggested by Stutterecker *et al. *is the more accepted explanation for such a location for the growth of a leiomyoma [12].

## Conclusion

Thorough radiographic imaging of sonographically diagnosed leiomyomata is important, especially for those which are large in size or present in an uncommon location. Several theories have been postulated regarding the origin of retroperitoneal leiomyomata; however, the exact etiology is still an unexplored issue that merits more investigation.

## Consent

Written informed consent was obtained from the patient for publication of this case report and any accompanying images. A copy of the written consent is available for review by the Editor in-Chief of this journal.

## Competing interests

The authors declare that they have no competing interests.

## Authors' contributions

AS wrote the manuscript and was directly involved in the patient care. BE performed the histological examination, diagnosis and the photo acquisition. NH was directly involved in the patient care and contributed to the review of the literature. HS was involved in the manuscript idea and drafting. TE provided general surgical input and critical reading of the manuscript. All authors read and approved the final manuscript.
